# The Joint Log-Lift Task: A Social Foraging Paradigm

**DOI:** 10.3389/fvets.2021.745627

**Published:** 2021-10-11

**Authors:** Jean-Loup Rault, Irene Camerlink, Sébastien Goumon, Roger Mundry, Marek Špinka

**Affiliations:** ^1^Institute of Animal Welfare Science, University of Veterinary Medicine, Vienna, Austria; ^2^ETH Zürich, Animal Physiology, Institute of Agricultural Sciences, Zürich, Switzerland; ^3^Platform Bioinformatics and Biostatistics, University of Veterinary Medicine, Vienna, Austria; ^4^Department of Ethology and Companion Animal Science, Czech University of Life Sciences, Prague, Czechia; ^5^Department of Ethology, Institute of Animal Science, Prague, Czechia

**Keywords:** affiliation, coordination, cooperation, joint action, prosocial, spontaneous, sociability, social learning

## Abstract

Behavioural cooperation is under intense research. Yet, popular experimental paradigms often employ artificial tasks, require training, or do not permit partner choice, possibly limiting their biological relevance. We developed the joint log-lift task, a social foraging paradigm in which animals have to jointly lift a log to each obtain a food reward. The task relies on an obligate strategy, meaning that the only way to benefit is to work jointly. We hypothesised that (1) animals learn to spontaneously solve the task, and that (2) kin and (3) more sociable individuals would engage more often together in the task and achieve greater success than non-kin and less sociable individuals, respectively. We presented the task to 8 groups of juvenile domestic pigs (*Sus scrofa domesticus*) in their home pen for 30 min daily. Over the course of 9 days, the pigs showed evidence of learning by progressively switching from individual to joint behaviours, leading to 68% (62 out of 91 pigs) spontaneously solving the task. Success was influenced by sociability, but not kinship. There were large differences in success among dyads, hinting at the possible role of social dynamics and inter-individual differences in the ability and/or motivation to solve the task. The joint log-lift task allows researchers to investigate spontaneous cooperative tendencies of individuals, dyads and groups in the home environment through *ad libitum* engagement with the apparatus. This ecologically relevant paradigm opens the way to investigate social foraging experimentally at large scale, by giving animals free choice about when and with whom to work jointly.

## Introduction

Cooperation, a behavioural strategy in which agents achieve a common goal through coordinated action (1), has been investigated in a variety of animal species ranging from mammals (2–5) and birds (6) to social arthropods (7–9). Some experimental paradigms have been particularly popular to investigate cooperation [reviewed in (10)], such as the “string pulling task” (or “loose-string paradigm”) employed in more than 160 bird and mammal species (11). Such approaches have provided insightful knowledge on the socio-cognitive abilities of various species. Nevertheless, the biological relevance of some tests has been questioned because they use artificial paradigms (12) or cognitively complex tasks (13). Furthermore, most experiments did not provide the opportunity for partner choice, neglecting social factors as an important facet of cooperation. Consequently, support is growing to investigate cooperation across animal species using ecologically-relevant contexts and species-specific paradigms (10).

We aimed to develop a task that would fulfil the requirements of being biologically relevant, intuitive enough to be solved spontaneously, and applicable to a group setting so that animals can choose their partner and work voluntarily. We developed the joint log-lift task (JLLT), in which two animals have to jointly lift a log to each obtain a food reward. Hence, the approach simulates a social foraging task. The task relies on an obligate strategy, meaning that the individuals only benefit if they work jointly and do not get a (lower) reward for performing the task individually, mimicking many conditions of cooperation found in nature when the only way to access a resource is to cooperate (14). Furthermore, the apparatus is designed so that it can be placed in the home environment and presented to a social group, thus allowing free partner choice and voluntary engagement with the apparatus.

There are conflicting findings on the importance of social factors on the propensity to cooperate. The kinship selection theory (15) was initially the predominant explanation, by cooperating with genetically related individuals to enhance one's inclusive fitness. However, cooperation between non-kin occurs based on alternative mechanisms such as reciprocity (16). In addition, factors at group level related to social dynamics or social relationships have been found to be important for cooperation [reviewed in (10)]. Unfortunately, relatively few studies used set-ups that allow partner choice in order to better understand the social factors involved in cooperation (10, 12, 17–20).

We tested domestic pigs (*Sus scrofa domesticus*) on the JLLT, as they are highly social animals that engage in cooperative behaviours such as coordinated nursing solicitation by piglets (21), nursing synchronisation (22), and occasional communal rearing (23). Pigs also consider social cues while foraging (24, 25). Their wild boar ancestors are capable of efficient temporal and spatial coordination while foraging, when cooperation prevails over competition (26). Foraging in pigs is typically done with the snout, rooting in the soil or under organic materials laying on the ground to find food items, and pigs possess a particular strong force in their snout to dig or lift (27). Hence, the JLLT solicits a biologically-relevant behaviour for pigs by asking them to lift a log.

We hypothesised that (1) pigs learn to spontaneously solve the task by working jointly, (2) kin would engage more often together in the task and achieve higher success than non-kin, and (3) more sociable individuals would engage more often in the task and achieve greater success than less sociable individuals.

## Methods

### Animals and Housing

Eight groups of mixed-sex young pigs (*N* = 91 pigs) of a commercial farm breed were studied from 7 to 9 weeks of age. Pigs were housed in 3.1 × 4.7 m partly-slatted floor pens, with a 3 × 1 m covered heated sleeping area, *ad libitum* access to feed through a multi-space feeder containing a commercial pig meal diet, and *ad libitum* access to water through four drinkers. They were provided with environmental enrichment in the form of straw, wood shavings in the sleeping area, and a small wood log hanging on a chain in the pen. Room temperature was recorded at noon on each testing day, with an average temperature of 17.3°C (range: 15.1–18.8°C).

### Joint Log-Lift Task Apparatus

**Figure 1:**
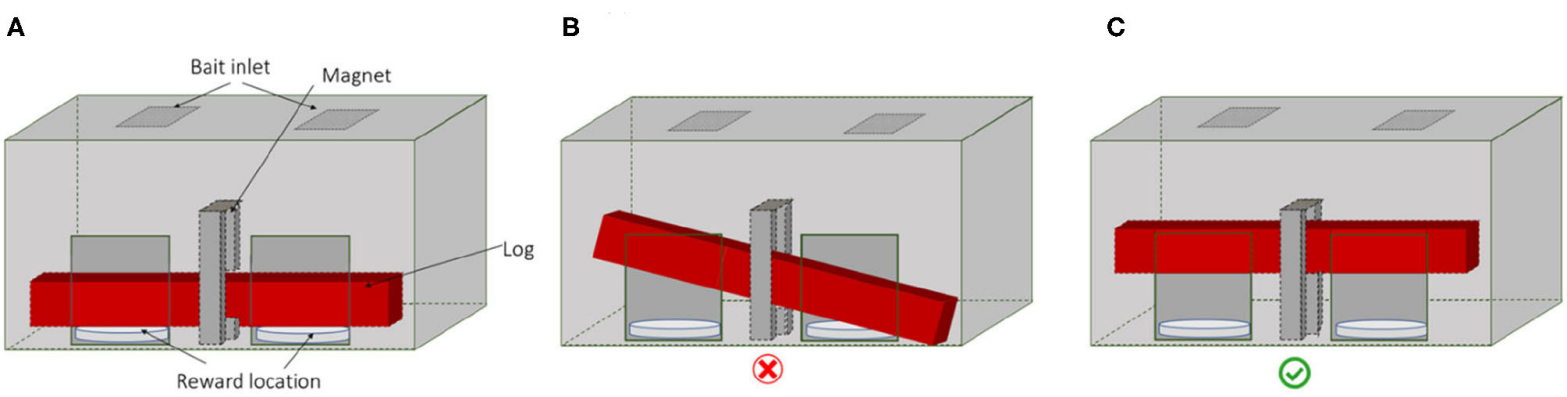
The apparatus in three positions: **(A)** log is down; **(B)** if only one pig lifts the log or if two pigs lift the log but unevenly or not high enough then the magnet does not hold the log and no reward is provided; **(C)** two pigs lift the log to the top magnet and food rewards are provided.

The testing apparatus requires that two animals lift up a wooden log simultaneously to each receive a food reward (Figure 1; see Supplementary Video). If the log is lifted up on both sides simultaneously a magnet holds the log in the upper position and a food reward is released on each side (Figure 1C). If only one individual lifts up the log, or if individuals lift the log on both sides but each to a different height, the log cannot fix to the magnet due to the inclination and the food rewards remain inaccessible (Figure 1B).

The test apparatus was 75 cm wide and 63 cm high and had two openings at the front, each measuring 15 × 25 cm (width × height) separated by 25.5 cm from each other. The sides were made of wood whereas the front was a 4 mm transparent Plexiglas to allow the animals and the camera to see the log moving. Two 33 cm round plastic food bowls were placed below each opening to receive the food rewards. The 70 cm long and 2 kg wooden log laid out horizontally in the apparatus, resting on the food bowls. A metal strip was attached in the middle of the top surface of the log to lock in with the magnet. The magnet was placed at a height of 30 cm in the middle of the apparatus above the middle of the log and held the log in place when the log was lifted high enough on both sides to come in contact with at least 50% of the magnet's surface area. The direction of movement of the log was guided by a metal rail in the centre that guided the log to move vertically. The magnet locked only when the log was lifted from both sides simultaneously to the required height.

### Habituation

A food preference test was conducted in each group prior to testing by offering to the group simultaneous choices between a total of eight heaps of 60 g of either chocolate-coated raisins, apples cut into 0.5 × 0.5 cm cubes, Solettis (salted sticks), and dried mealworms, with two heaps per feed type. Preferences were ranked based on which heap of feed items was depleted first, on group level. Across the eight groups of pigs, 89% of the first depleted feed items were the apple pieces, whereas 75% of the second depleted feed items were the chocolate raisins, with the rest divided between the salted sticks and dried mealworms.

Thereafter, on 2 consecutive days prior to the test, the apparatus was placed in each pen for 10 min per day while the log was locked up on the magnet. This way, pigs could explore the apparatus and discover that it contains food rewards (apple pieces mixed with chocolate raisins) in the food bowls, which were rebaited with small portions when pigs had finished consuming the food rewards.

### Testing Days

Tests took place over 9 consecutive days with 30 min daily testing sessions, starting from when pigs were 7 weeks of age. Tests were carried out between 09:30 and 15:00 h. Pigs were marked on their back with an animal marker spray for individual recognition and remarked, when necessary, after the test session of that day was completed. The test order of the groups were randomised across the testing days. Before the test, the experimenter stepped into the pen and set up a camcorder to video record the test from the front view of the apparatus. Entering the pen always woke up the pigs in the few occasions that they were asleep prior to testing, ensuring that they were aware of the start of the testing session. The test was started by placing the testing apparatus, with the log down, into the pen resting on the floor along the pen's door side. The pigs could then freely interact with the apparatus for 30 min per group. A single observer noted the timing, frequency and type of interactions with the apparatus according to an ethogram (Table 1) and the identities of the pigs displaying the behaviour. The observer stood outside the pen and behind the apparatus. When two pigs successfully lifted the log so that it remained fixed to the magnet, emitting a soft click sound, they received a food reward within 2 s of success with the experimenter manually placing a small amount (5–10 g) of small cut apple pieces (0.5 × 0.5 × 0.5 cm, Jonagold apples) mixed with chocolate raisins in their food bowl from two openings on the top of the apparatus. The apparatus was reset immediately after the pigs had consumed the reward. Food was not present in the bowl until after the pigs had succeeded.

**Table 1:** Ethogram to record the behavioural interactions with the joint log-lift task apparatus.

**Behaviour**	**Definition**
Touch	A pig makes physical contact with the log with its snout
Attempt alone	One pig raises the log from one side alone, i.e., without another pig lifting on the other side
Attempt together	Two pigs jointly raise the log but do not manage to get it locked to the magnet at the top, because the movement is too uncoordinated or quick
Successful lift	Two pigs lift the log and successfully lock it to the magnet so that the log remains in the upper position

On the first 3 testing days, the apparatus was pre-baited (i.e., food placed in the reward location) to increase motivation so that the pigs could smell the food reward below the log but not reach it unless the log was lifted successfully by both pigs. Hence, one pig was unable to reach the food rewards alone. There were occasionally pigs who displaced others and therefore, to avoid this, we stopped pre-baiting after day 3 and rewarded the pair of pigs immediately after success to avoid free-riding. Therefore, on the remaining days, the food bowls were not pre-baited unless the group had not been successful in the previous day in which case we pre-baited the apparatus to maintain the pigs' interest in the task. This was the case for group 1 on test days 6, 7, and 8; group 3 on day 8; and group 7 on days 7 and 9.

### Group Composition and Kinship

After weaning at 4 weeks of age, pigs were either kept with their original littermates (“littermate” groups; *n* = 4 groups), or a new group was composed by mixing four different litters (“mixed” groups; *n* = 4 groups) to generate variation in group composition. In the littermate groups, the number of pigs per group varied between 10 and 14, based on initial litter size and as the initial group composition (i.e., litter) was kept intact. The mixed groups were matched to have a similar variation in group size as the littermate groups, with an average group size of 12 (Std. dev.: 1.825; range: 10–14) pigs in the littermate groups and 11 (Std. dev.: 0.816; range: 10–12) pigs for the mixed groups. Due to routine practices at the farm, piglets were fostered-off only when the number of piglets exceeded the number of functional teats of the sow and within 72 h after birth. There were 15 fostered pigs of which four were in the littermates groups and 11 in the mixed groups. The only four fostered pigs in the “littermate” groups were considered as kins of the other pigs in their litter of adoption, because they spent most of their life up to weaning with them, although they were not genetically related, but in pigs familiarity prevails over genetic relatedness (28). None of the pigs were lost-to-follow-up (i.e., removed from the study, e.g., due to health reasons), so group composition remained unchanged throughout the experiment.

The variable “kinship” indicated for each possible dyad combination whether the individuals within a dyad interacting with the apparatus were littermates or not. The effect of kinship was nested within group composition, as pigs in littermate groups were all kin whereas pigs in mixed groups could interact with kin and non-kin. Depending on the size of the mixed groups (between 10 and 12 pigs originating from four litters), an animal could have between 77.8 and 88.9% of unrelated animals in the mixed groups, i.e., non-kin.

### Sociability

Sociability was recorded for 4 h daily for 5 days (3 days in the week after weaning and once weekly for the following 2 weeks). The following behaviours were recorded: nose-to-nose contact, nosing in proximity, nosing head, nosing body, allo-grooming, ano-genital nosing, exploring together, social play, lying together, mounting, agonistic behaviour, oral manipulation, belly nosing, other behaviour, or being out of sight, with the ethogram detailed in (29). Three observers, using the app Animal Behaviour Pro (version 1.4.4., Animal Behaviour Pro) installed on iPads, recorded the behaviour using 5-min scan sampling for each individual, resulting in 48 scans per animal per day (240 scans per animal in total). Intra- and inter-observer reliability was tested as intraclass correlation coefficient in R (version 3.6.3; R Core Team). The intra-observer reliability reached values of over 96.3% and the inter-observer reliability reached an agreement of 83.5%. The frequencies from all observation days were summed by behaviour by animal, and thereafter expressed as a proportion of the total number of scans. We created the variable “sociability” as the sum of the proportion of scans when an individual was observed initiating nose-to-nose contact, nosing in proximity, nosing head, nosing body, allo-grooming, exploring together and social play. As discussed in Camerlink et al. (29) ambiguous or potentially negative social behaviours such as ano-genital nosing, belly nosing, mounting, agonistic behaviour, and oral manipulation, or inactive behaviour like lying together were discarded from the sociability score. This resulted in one score per individual based on the frequency of the socio-affiliative behaviours initiated toward group members. We then calculated sociability scores for all possible dyads in the group by averaging the sociability of both individuals associating as a dyad in the task, i.e., for the joint behaviours Attempt together and Successful lift.

### Evidence of Understanding of the Social Nature of the Task

To determine whether the pigs showed evidence of understanding the need for a partner to solve the task, video footage of the six most successful groups on the last testing day (Day 9), when most pigs had learned how to solve the task, was analysed. We recorded lifting alone in presence or absence of a partner facing the second opening of the apparatus. We also recorded whether the lift was synchronised, defined as two pigs performing a Successful lift by lifting the log starting out from a horizontal position, or non-synchronised, defined as a Successful lift after one pig lifted the log up completely on its side (such that the log is in a diagonal position) followed by the second pig lifting the log on the other side.

### Statistical Analysis

Hypothesis 1 regarding learning of the task was tested based on the changes in behaviour over time (test days). Hypothesis 2 regarding kinship was tested based on the predictor variables “group composition” (littermates vs. mixed) for all behaviours, and “kinship” for the joint behaviours (Attempt together and Successful lift) as the variable kinship depended on dyadic associations. Hypothesis 3 regarding sociability was tested based on the predictor variable “sociability” for the joint behaviours (Attempt together and Successful lift) as this variable also depended on dyadic associations.

The frequencies of the four apparatus-directed behaviours “Touch,” “Attempt alone,” “Attempt together,” and “Successful lift” were analysed in separate models, models 1–4 as described below.

### Statistical Analyses of Touch and Attempt Alone

The frequencies at which each individual touched the apparatus (“Touch”; model 1) and attempted to lift the log alone (“Attempts alone”; model 2) were analysed with Generalised Linear Mixed Models [GLMM; (30), with Poisson (model 1) or Negative binomial (model 2) error structures, and log link function (31)]. The key predictor variables were test day (1 to 9), group composition (mixed vs. littermate), sex (male vs. female), and the interaction between test day and group composition or sex if significant, as fixed effects. We also included fostering (yes vs. no) and the individuals' birth weight to account for possible variation related to early life experience and dominance. To avoid pseudo-replication we included random intercept effects for the individual, the litter from which it originated, the group, and the day of test. In order to avoid an “overconfident” model, to keep type I error rate at the nominal level of 0.05, and to model the effect of fixed effects terms potentially varying between, for instance, groups or dyads, we included all theoretically identifiable random slopes (32, 33); see Supplementary Table 1 for an overview of all models fitted and the random slopes included). As an overall test of the effects of the key predictors, and to avoid cryptic multiple testing (34), we compared this full model with a null model lacking these fixed effects but being otherwise identical. The full and respective null models were compared by means of a likelihood ratio test (35) and the significance of individual fixed effects determined by dropping them one at a time R-function drop1; (33). The full-null model comparisons were significant (model 1: χ^2^ = 23.073, df = 4, *P* < 0.001; model 2: χ^2^ = 20.566, df = 4, *P* < 0.001), indicating that the test predictors as a collective had a clear impact on those response variables.

### Statistical Analyses of Attempt Together and Successful Lift

The frequencies of dyadic attempts to lift the log together (“Attempt together”; model 3) and to successfully lift together (“Successful lift”; model 4) were analysed with GLMMs with Poisson error structure and log link function. The predictor variables were test day (1 to 9), group composition (mixed vs. littermate), kinship (whether the two members of a dyad originated from the same litter or not), the interactions between test day and group composition or kinship, the average sociability score per dyad, and the sex combination of the dyad (female-female, female-male, or male-male). We included random intercept effects for the pen, the dyad, the two members of the dyad, the two litters from which they originated, and test day nested within pen. Again, we included all theoretically identifiable random slopes (Supplementary Table 1). As animals could associate with various partners, we included all possible dyads and allocated “0” for dyads that never interacted. We compared this full model with a null model lacking the fixed effects but being otherwise identical. The full-null model comparisons were not significant for the test predictors as a whole (model 3: χ^2^ = 3.945, df = 5, *P* = 0.557; model 4: χ^2^ = 7.403, df = 5, *P* = 0.192), but significant main effects appeared in a reduced model after removal of the non-significant interactions (see results).

### General Aspects of the Statistical Analysis

Data were fitted with models in R (version 3.6.3; R Core Team) using the function glmer of the package lme4 [version: 1.1-21; (36)]. We confirmed model stability by dropping levels of random effects one at a time and comparing the estimates derived for models fitted to those subsets with those obtained for the model for the full data set. We determined 95% confidence intervals of model estimates and fitted values by means of parametric bootstraps (N = 1,000 bootstraps; function bootMer of the packages lme4). None of the models was overdispersed (dispersion parameters model 1: 0.988; model 2: 0.715; model 3: 0.605; model 4: 0.352), and collinearity was no issue. Samples sizes are reported in Supplementary Table 1.

## Results

### Behavioural Interactions With the Apparatus

**Figure 2:**
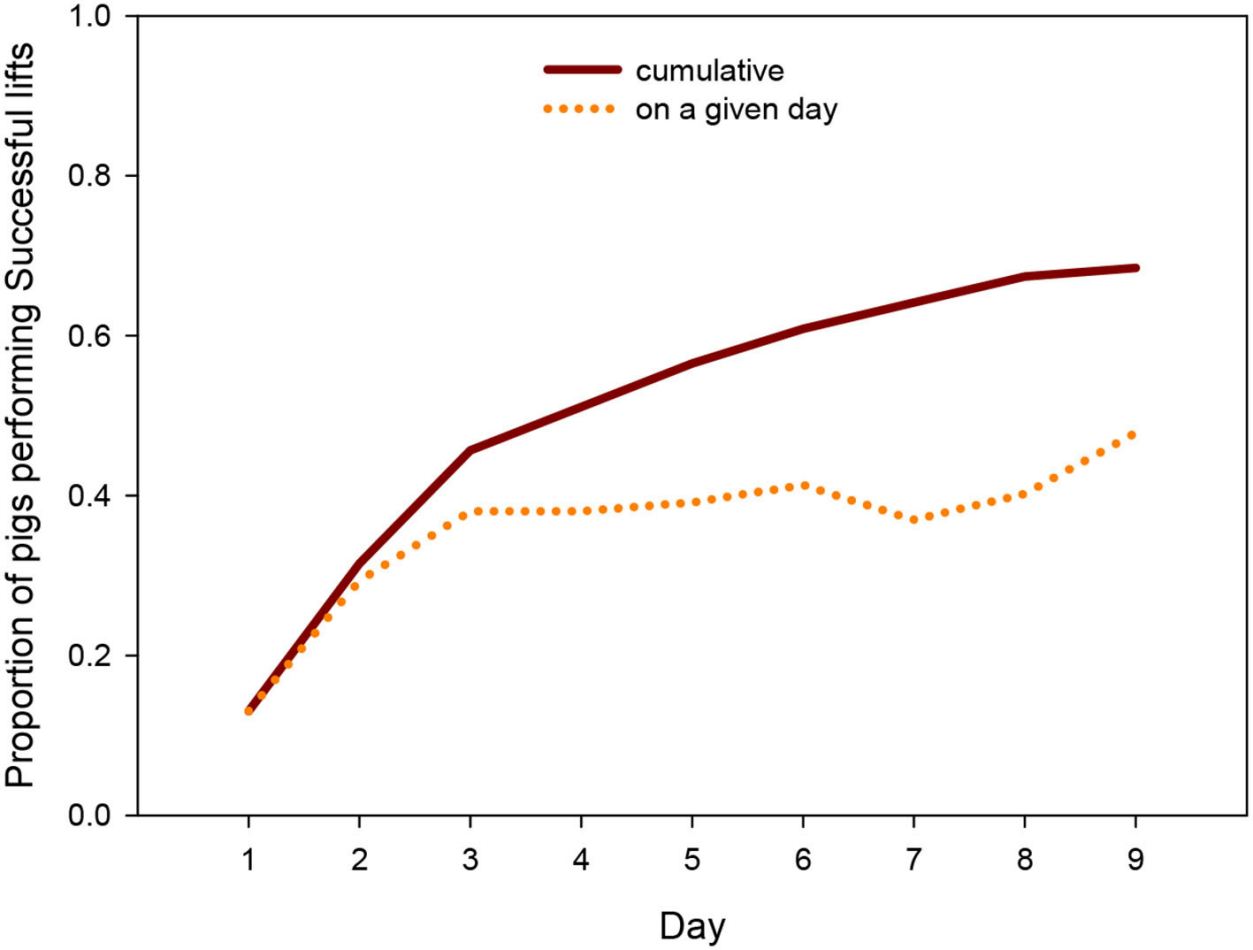
Proportion of pigs per day and cumulative proportion that successfully lifted the log across days.

Across the days, 68% (*N* = 62) of the animals performed at least once a Successful lift, i.e., solved the task through a joint action with another group member. The proportion of successful individuals on a given day rose quickly until day 3 and more slowly thereafter (Figure 2), with almost half of the pigs being successful on the last day (“on a given day” count) and two thirds of the pigs having succeeded at least once over the course of the 9 days (“cumulative count”).

**Figure 3:**
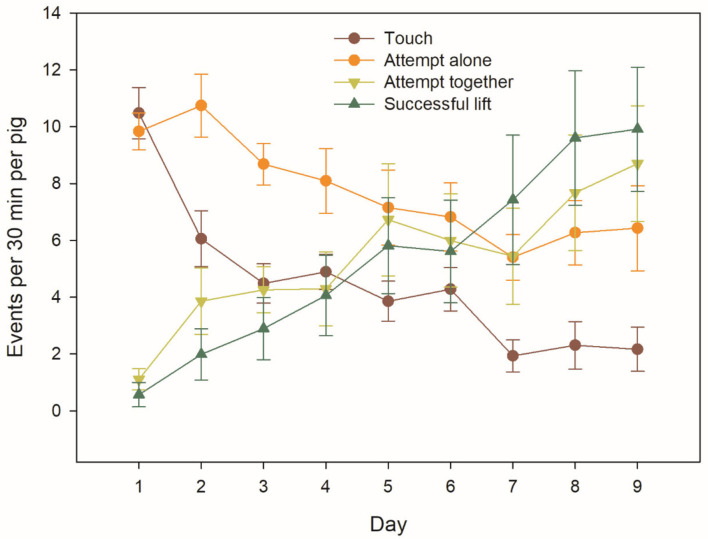
Behavioural interactions with the apparatus over the testing days, based on group averages (*n* = 8) of individual pig behaviour. The bars represent the standard errors.

In total, there were 2261 Successful lifts over the 9 test days (Figure 3). Nevertheless, there was a large variation among the 91 individual animals in the number of Successful lifts. The top nine individuals from four different groups accounted for 50.5% of all Successful lifts, and there were large differences in the quantiles of Successful lifts across the 91 animals (minimum = 0, 1st decile = 0, 1st quartile = 0, median = 3, 3rd quartile = 64, 9th decile = 179 and maximum = 319 Successful lifts).

The frequency of behavioural interactions with the apparatus averaged 27.0 (min: 21.7, max: 34.4) interactions per pig and 30-min test across days, when all behaviours were summed up. Individual behaviours (Touch and Attempt alone) were progressively replaced by joint behaviours (Attempt together and Successful lift) (Figure 3).

### Evidence of Learning

**Figure 4:**
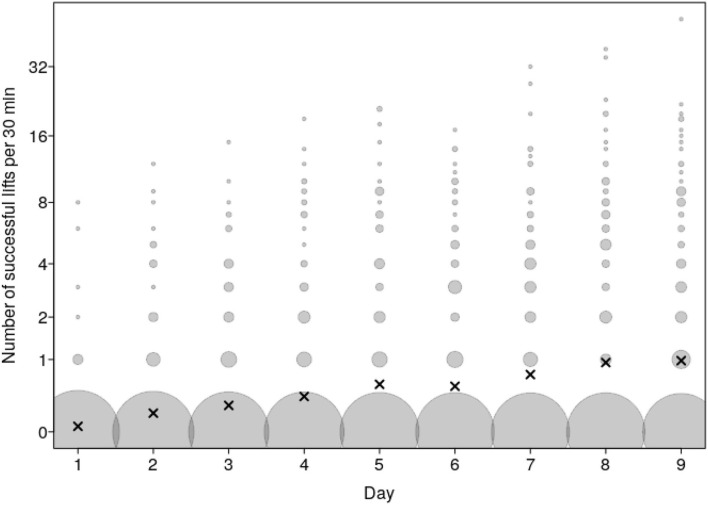
Number of Successful lifts as a function of test day. Dots show the number of Successful lifts per test day and dyad whereby the area of the dots depicts the number of cases per combination of test day and number of Successful lifts (range: 1 to 468). The laying crosses depict the mean per day. A value of 0 was allocated to any of the potential dyadic combination of pigs in a group that did not perform a Successful lift on that day; hence the large number of 0 values.

The frequency of Successful lifts increased over the course of the test days (*P* = 0.004; Figures 3, 4 and Supplementary Table 2). The frequency of Attempts together also tended to increase over the course of the test days (*P* = 0.08; Figure 3 and Supplementary Table 3). The frequency of Attempts alone and Touches differed or tended to differ, respectively, according to the interaction of test day and group composition (see below).

### Group Composition and Kinship

The frequency of Successful lifts and Attempts together did not significantly differ according to group composition or kinship (Supplementary Tables 2, 3, respectively).

**Figure 5:**
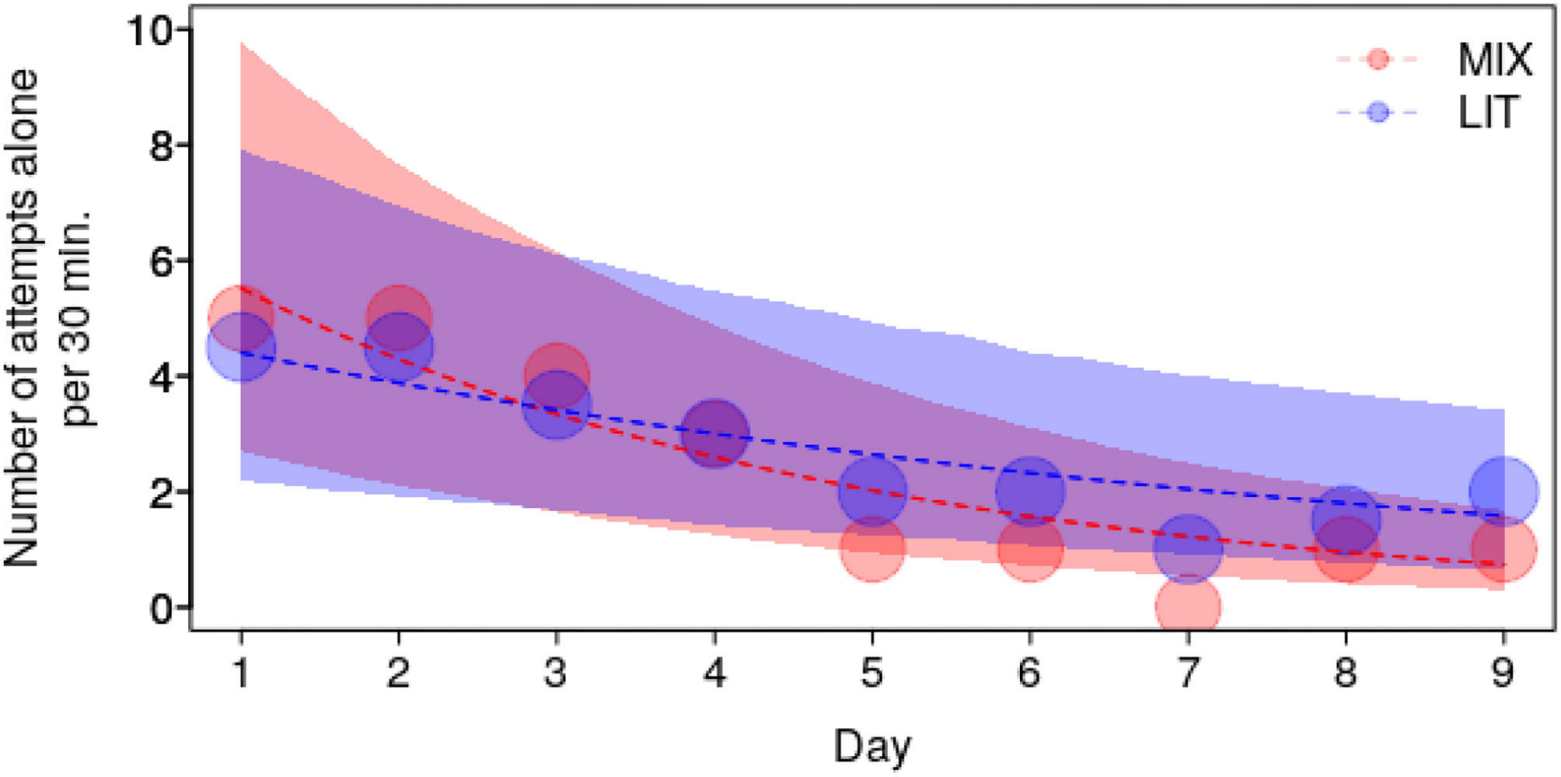
Frequency of Attempts alone as a function of test day, for the two group compositions with mixed groups in light red (MIX) and littermate groups (LIT) in dark blue. Dots show the median number of Attempts Alone per test day whereby the area of the dots depicts the number of counts (range: 43 to 48). The dashed lines and the shaded polygons depict the fitted model and its confidence limit.

The frequency of Attempts alone decreased according to the interaction of group composition and test day (*P* = 0.03; Figure 5 and Supplementary Table 4), whereby it decreased over the course of the test days in both group compositions but was steeper in the mixed groups as compared to the littermate groups (MIX: −0.65+0.11, z = −5.71, *P* < 0.001; LIT: −0.33+0.1, z = −3.46, *P* = 0.001). The frequency of Touches also tended to decrease according to the interaction of group composition and test day (*P* = 0.06; Supplementary Table 5) whereby the decrease over the course of the testing days also tended to be steeper in mixed groups as compared to the littermate groups.

### Sociability

The frequency of Successful lifts increased with greater sociability score (*P* = 0.03; Supplementary Table 2). The frequency of Attempts together did not significantly differ according to sociability (Supplementary Table 3).

### Dyads

**Figure 6:**
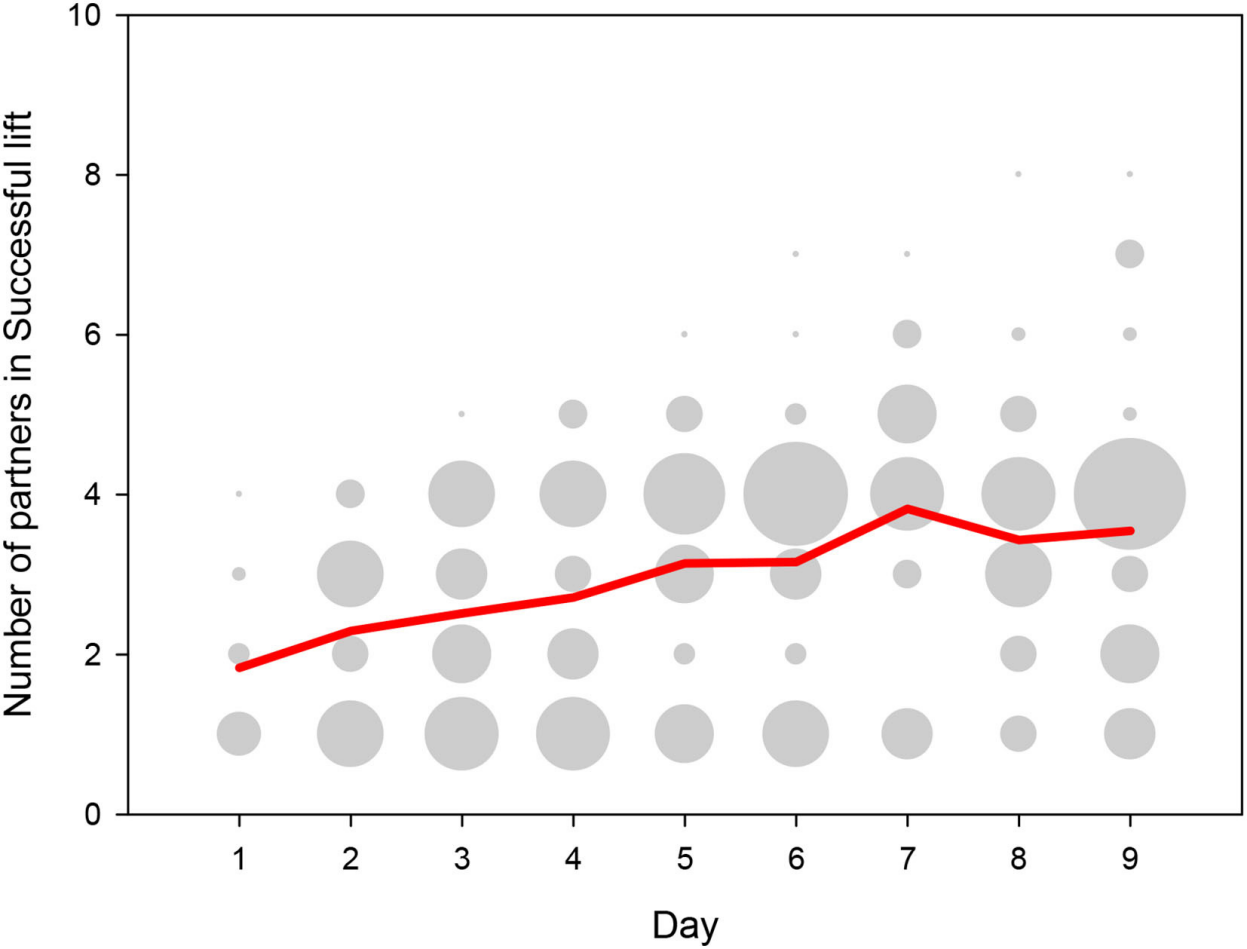
Number of partners with whom a pig successfully lifted the log, depicted for those pigs that lifted the log at least once on a given day. The y-axis shows the number of partners per test day for a given pig whereby the dot size depicts the number of pigs that lifted for that specific number of partners. The red line depicts the mean number of partners across days.

Over the days, successful pigs gradually paired up with an increasing number of different partners (Figure 6), and over the last 3 days several pigs associated with 6 or more different partners. There was a large variation in the total number of success between dyads, indicating that the dyadic associations were strongly non-random. The average number of Successful lifts was 0.5 per 30 min daily session and 4.7 per dyad over the course of the 9 test days, but the distribution was highly skewed between dyads. On the one hand, only 26% (124 out of 479 possible) dyadic combinations of pigs successfully lifted together, in spite of the fact that pigs had the choice of partners within their group over the duration of the experiment. On the other hand, some of these dyads were highly successful, with 39 dyads with 20 or more Successful lifts over the nine sessions, 12 dyads with 50 or more Successful lifts, and even three dyads with over 100 Successful lifts.

### Evidence of Understanding of the Social Nature of the Task

From the subset of data analysed for the six most successful groups on the last testing day, out of 462 occurrences of lift alone, 434 (93.9%) were lifts in the presence of a partner facing the second opening of the apparatus, and the rest in absence of a partner. Of 448 lift successes, 112 (25%) were synchronised, and the rest non-synchronised.

## Discussion

Pigs solved the JLLT spontaneously, progressively switching from individual to joint behaviours, with almost half of the pigs being successful on the last day and two thirds of the pigs having succeeded at least once over the course of the 9 days. The JLLT relies on an obligate strategy, meaning that the individuals only benefit if they engage in the task jointly and do not get a (smaller) reward for performing the task individually. Thus, the task is more demanding than other situations where succeeding alone is possible (12, 14). This task can easily be adapted to socially foraging species in an ecologically-relevant manner, for instance for primates that may attempt to lift the log with their hands, elephants with their trunks, or other suids with their nose. The principle of the task can be also used for construction of a device, with a modified mechanical design, for species that use other specific motor skills during foraging, such as pulling a branch or scratching the ground. We posit that such a device could be designed for various species in which individuals often forage synchronously in close vicinity of each other and frequently use a specific motor pattern to access a food source.

Our findings do not provide evidence about the specific cognitive mechanisms behind the successful joint action of the pigs at the apparatus. We presume that the task may reflect cooperation, based on the definition by Noë (1) of “a behavioural strategy in which agents achieve a common goal through coordinated action.” However, the term “cooperation” remains debated (37), from simple definitions such as two or more animals acting simultaneously or sequentially to solve a problem (12, 38), to much stricter definitions requiring the capacity to understand the role of the partner and to share intentions (39). Consequently, terms such as similarity or independent cooperation (38), coordination (40), collaboration (41), and intentional cooperation (38) have been proposed to distinguish between different types of joint actions (i.e., “cooperation” in the wide sense of the term). Even within the same act of cooperation, the participating individuals may differ in what cognitive mechanisms they employ (42). The mechanisms involved in solving the JLLT remain to be elucidated.

The animals were able to solve the JLLT spontaneously. Spontaneous cooperation [e.g., (5, 18, 20, 43, 44)] has been much less studied than instrumental cooperation when animals are trained at the task (12, 13). Spontaneous learning was likely facilitated by the design of the apparatus, enticing an elementary natural foraging behaviour. It also allowed researchers to circumvent the need for prior separate individual training, which can be problematic when testing cooperation that requires coordination rather than the mere combination of two individuals having learnt to succeed independently (12, 45). Kune Kune free-ranging pigs failed to spontaneously solve the JLLT when presented with it for 18 trials over 3 sessions in selected pairs, but the Kune Kune pigs succeeded at it after training (46). Pigs in the present study managed to successfully solve the JLTT when presented with the task in the home environment and in their group with *ad libitum* trials over 30-min for 9 days, allowing for more trial-and-error opportunities than this other study. The success then depended solely on coordinating the action in time with a suitable partner within the group. The progressive change in behaviour from single to joint actions supports that the pigs learned how to solve the task, as experience is often necessary for cooperation (47). The time from the first exposure to the apparatus to solving the task was among the shortest ever recorded in studies with other tasks including primates (48), social canids (49), and parrots (50).

The highly skewed distribution of success across dyads indicates that mutual free choice of partners may be a key aspect for success. More sociable pigs were found to be more likely to perform Successful lifts, although their engagement in Attempts together did not differ, suggesting greater proficiency at the task in more sociable pigs. This possibly reflected social tolerance as a factor conducive to cooperation in other species (18, 51). Offering the task at group level also allowed us to test animals in their home environment, hence without disturbance (e.g., handling, novel environment), and with free-choice about when and with whom to work jointly. Similarly chimpanzees free to interact with an apparatus in their home enclosure selected their preferred cooperation partner to solve the task (5). Testing in the home environment allows accounting for social and other contextual features that may have core influences on the propensity of animals to interact with others (26, 45, 52, 53).

Kin were however not more likely to engage or succeed in the task than non-kin. The only difference was that pigs in mixed groups reduced their attempts alone quicker than pigs in littermate groups, but with no difference in attempting together or successful lifts. Pigs are able to recognise familiar individuals from a young age (28) and can form affiliation with kin (54) and non-kin (55). Nevertheless, pigs in the wild live in groups of genetically related females with little fission-fusion dynamics (56), possibly explaining their lack of kin discrimination. Kinship through inclusive fitness is a common explanation for cooperation, but cooperation can also operate based on reciprocity (16). The highly skewed distribution of lifts across the dyads indicates that successful pigs appeared to form preferential dyadic associations to solve the task. Thus, pigs may learn to associate with more proficient partners over the course of their trials, or based on their affiliative preferences (55).

The majority of pigs were highly motivated to work to access the high-value food reward, despite having *ad libitum* access to feed. Pigs show a high motivation for foraging when provided the opportunity (57). The number of successful individuals on a given day rose until day 3, the last day in which the apparatus was pre-baited, but pigs maintained interest thereafter, and whether pre-baiting the apparatus heightens interest requires further research. Given that pigs became increasingly successful over the days but the total number of interactions with the apparatus remained similar, it appears that a steady motivation was driving the learning process that was faster in some individuals, slower in others, and failed (within the given timeframe) in yet other individuals. Although the task theoretically allows each pig to engage with the apparatus when they want and with whom they want, a third of the pigs did not succeed, which suggests that either they were unable to solve the task, would have needed longer, or possibly were unable to access the apparatus during the relatively short testing sessions due to the monopolisation of the apparatus by other pigs. These limitations could be solved by providing access to the apparatus for longer test sessions and over a longer time period.

The underlying cognitive mechanism of the JLLT remains to be elucidated. One possibility is that pigs are using their capability to synchronise their behaviour (58) to achieve the time-coordinated joint action at the apparatus. It has been shown both through modelling and experimental research that synchronisation in pairs could be achieved without explicit communication and that it can promote joint performance (59, 60). However, the pigs in this study showed synchronised lifting in only one quarter of the coded test sessions. Nevertheless, other mechanisms can facilitate joint action or coordination (12), including social facilitation (61). Success in the JLLT task could also result from chance associations of individuals acting simultaneously, as seen in other social foraging tasks [e.g., (20)]. At least, pigs lifted in a large majority of cases (94%) when another partner was present at the other hole, rather than when no partner were present. Further research should use appropriate controls to test whether pigs take account of the partner's presence and behaviour [e.g., (46, 62)].

## Conclusions

The feasibility of the JLLT makes it a versatile tool for experimental investigations of social foraging at large scale, by giving animals free choice about when and with whom to work jointly. This approach offers the possibility to study the role of partner choice, group social dynamics, personality, and proficiency in the task (ratio of success to failure) on the performance of different species of animals. At the same time, the variability of success in the JLLT between individuals and dyads within groups in the current study indicates that the task may be a valuable paradigm to assess various factors that affect social associations at different levels of animal social organisation.

## Data Availability Statement

The datasets presented in this study can be found in online repositories. The names of the repository/repositories and accession number(s) can be found at: http://dx.doi.org/10.17632/4vb5xnm393.1.

## Ethics Statement

The animal study was reviewed and approved by Animal Ethics Committee of the University of Veterinary Medicine, Vienna, Austria (Research Protocol No. ETK-95/06/2019).

## Author Contributions

J-LR, IC, MŠ, and SG designed the apparatus, wrote the manuscript, and participated in data analysis and interpretation. J-LR and IC conceived and designed the study. IC carried out the experimental work. RM carried out the statistical analyses. All authors gave final approval for publication.

## Funding

MŠ was supported by grant 20-26831S from the Czech Science Foundation and grant MZE-RO0718 from the Ministry of Agriculture of the Czech Republic.

## Conflict of Interest

The authors declare that the research was conducted in the absence of any commercial or financial relationships that could be construed as a potential conflict of interest.

## Publisher's Note

All claims expressed in this article are solely those of the authors and do not necessarily represent those of their affiliated organizations, or those of the publisher, the editors and the reviewers. Any product that may be evaluated in this article, or claim that may be made by its manufacturer, is not guaranteed or endorsed by the publisher.
