# Supplementary material for: The Joint Log-Lift Task: A Social Foraging Paradigm
Source: Front Vet Sci. 2021 Oct 11;8:745627. doi: 10.3389/fvets.2021.745627 (PMC8542970; doi:10.3389/fvets.2021.745627)
Supplement: Supplementary file 2 [file Data_Sheet_1.docx]

**Table S1.** Overview of the models fitted

| model | Response | Fixed effects^(1)^ | Random effects^(2)^ | Error structure/ link function | N |
| --- | --- | --- | --- | --- | --- |
| 1 | Touch | test day * (Sex + Group compos.) + fostered + birth wgt | (1 + test day\|Indiv) +  (1 + birth wgt + fostered + Sex * test day\|\|litter) +  (1 + birth wgt + fostered + Sex * test day\|\|Pen) +  (1 + birth wgt + fostered + Sex\|day.in.gr), | Poisson/ log link | 819 total;  91 Indiv;  72 test day;  8 Pen;  8 litter |
| 2 | Attempt alone | test day * (Sex + Group compos.) + fostered + birth wgt | (1 + test day\|Indiv) +  (1 + birth wgt + fostered + Sex * test day\|\|litter) +  (1 + birth wgt + fostered + Sex * test day\|\|Pen) +  (1 + birth wgt + fostered + Sex\|day.in.gr) | Negative binomial/ log link | 819 total;  91 Indiv;  72 test day;  8 Pen;  8 litter |
| 3 and 4 | Attempts together  and  Successful lift | test day * (Group compos. + kin) + sociab + sex.comb | (1 + test day * kin.yes + sociab + sex comb\|\|Pen) +  (1 + kin.yes + sociab + sex comb\|\|test day) +  (1 + test day\|\|dyad) +  (1 + test day + sociab + sex comb\|\|Indiv1) +  (1 + test day + sociab + sex comb\|\|Indiv2) +  (1 + test day * kin.yes + sociab + sex comb\|\|sow1) +  (1 + test day * kin.yes + sociab + sex comb\|\|sow2) | Poisson/ log link | 4311 total;  479 dyad;  83 Indiv2;  83 Indiv1;  72 test day;  8 sow2;  8 sow1;  8 Pen |

^(1)^ group composition, sex, fostered, and sex combination (‘sex comb’) were dummy coded with 'mixed', 'female', 'not fostered' and 'FF' being the respective reference categories; test day, birth weight (‘birth wgt’), and sociability (‘sociab’) were z-transformed; an asterisk denotes an interaction and all terms it encompasses

^(2)^ factors were generally represented by centered dummy variables whereby sex comb stands for two such variables; we use the lme4 annotation here, *i.e.*, (1+x|g) refers to a random intercepts effect of g, a random slope of x within g and also the correlation between them whereas (1+x|g) means the same but without the correlation parameter.

**Table S2.** Results of the reduced model for the frequency of Successful lifts as the response (model 4, lacking the non-significant interactions; indicated are estimates, together with standard errors, confidence limits, and significance tests). Statistically significant results appear in bold.

| Term | Estimate | SE | lower Cl | upper Cl | χ^2^ | df^(1)^ | P |
| --- | --- | --- | --- | --- | --- | --- | --- |
| Intercept | -5.745 | 1.062 | -8.406 | -4.183 |  |  | ^(1)^ |
| **test day^(2)^** | **1.168** | **0.317** | 0.664 | 1.714 | **8.178** | **1** | **0.004** |
| group compos.^(3)^ | 0.447 | 1.164 | -1.696 | 2.891 | 0.299 | 1 | 0.584 |
| kin^(4)^ | -0.137 | 0.462 | -1.521 | 1.132 | 0.128 | 1 | 0.720 |
| **sociab.^(5)^** | **0.268** | **0.588** | -0.893 | 1.439 | **4.487** | **1** | **0.034** |
| sex.combFM^(6)^ | -1.940 | 0.819 | -3.735 | -0.432 | 3.238 | 2 | 0.198 |
| sex.combMM | -1.100 | 1.343 | -4.059 | 1.517 |  |  |  |

^(1)^ not indicated because of having a very limited interpretation

^(2)^ z-transformed to a mean of zero and a standard deviation (sd) of one; mean and sd of the original variable were, 5.000 and 2.582, respectively

^(3)^ dummy coded with 'mixed' being the reference category

^(4)^ dummy coded with 'not kin' being the reference category

^(5)^ z-transformed to a mean of zero and a standard deviation (sd) of one; mean and sd of the original variable were, 9.712 and 2.528, respectively. This p-value should be interpreted cautiously as the confidence interval is rather wide.

^(6)^ dummy coded with FF being the reference category; the indicated test refers to the overall effect of the factor

**Table S3.** Results of the reduced model for the frequency of Attempts together as the response (model 3, lacking the non-significant interactions; indicated are estimates, together with standard errors, confidence limits, and significance tests).

| term | Estimate | SE | lower Cl | upper Cl | χ^2^ | df | P |
| --- | --- | --- | --- | --- | --- | --- | --- |
| intercept | -4.072 | 0.808 | -5.919 | -2.576 |  |  | ^(1)^ |
| test day^(2)^ | 0.247 | 0.133 | -0.021 | 0.524 | 3.015 | 1 | 0.083 |
| group compos.^(3)^ | 0.191 | 0.966 | -1.826 | 2.257 | 0.038 | 1 | 0.845 |
| kin^(4)^ | -0.285 | 0.293 | -1.065 | 0.434 | 0.819 | 1 | 0.366 |
| sociab.^(5)^ | 0.042 | 0.443 | -0.843 | 0.956 | 0.008 | 1 | 0.929 |
| sex.combFM^(6)^ | 0.046 | 0.376 | -0.721 | 0.884 | 0.126 | 2 | 0.939 |
| sex.combMM | 0.006 | 0.713 | -1.439 | 1.480 |  |  |  |

^(^1) not indicated because of having a very limited interpretation

(2) z-transformed to a mean of zero and a standard deviation (sd) of one; mean and sd of the original variable were, 5.000 and 2.582, respectively

(3) dummy coded with 'mixed' being the reference category

(4) dummy coded with 'not kin' being the reference category

(5) z-transformed to a mean of zero and a standard deviation (sd) of one; mean and sd of the original variable were, 9.712 and 2.528, respectively

(6) dummy coded with FF being the reference category; the indicated test refers to the overall effect of the factor

**Table S4.** Results of the full model for the frequency of Attempts alone as the response (model 2, lacking the non-significant interaction; indicated are estimates, together with standard errors, confidence limits, and significance tests). Statistically significant results appear in bold.

| term | Estimate | SE | lower Cl | upper Cl | χ^2^ | df | P |
| --- | --- | --- | --- | --- | --- | --- | --- |
| intercept | 0.725 | 0.414 | -0.123 | 1.505 |  |  | ^(1)^ |
| test day^(2)^ | -0.649 | 0.114 | -0.876 | -0.432 |  |  | ^(1)^ |
| group compos.^(3)^ | 0.267 | 0.494 | -0.716 | 1.165 |  |  | ^(1)^ |
| sex^(4)^ | -0.010 | 0.304 | -0.635 | 0.660 | 0.001 | 1 | 0.975 |
| fostered^(5)^ | -0.090 | 0.455 | -1.050 | 0.796 | 0.039 | 1 | 0.843 |
| **birth wgt.^(6)^** | **0.619** | **0.177** | **0.273** | **0.978** | **10.662** | **1** | **0.001** |
| **test day:group compos.** | **0.318** | **0.144** | **0.036** | **0.601** | **4.921** | **1** | **0.027** |

^(1)^ not indicated because of having a very limited interpretation

^(2)^ z-transformed to a mean of zero and a standard deviation (sd) of one; mean and sd of the original variable were, 5.000 and 2.584, respectively

^(3)^ dummy coded with 'mixed' being the reference category

^(4)^ dummy coded with female being the reference category

^(5)^ dummy coded with 'no' being the reference category

^(6)^ z-transformed to a mean of zero and a standard deviation (sd) of one; mean and sd of the original variable were, 6.873 and 1.538, respectively

**Table S5.** Results of the full model for the frequency of Touches as the response (model 1; indicated are estimates, together with standard errors, confidence limits, significance tests, as well as minimum and maximum of model estimates obtained after dropping levels of random effects one at a time).

| term | Estimate | SE | lower Cl | upper Cl | χ^2^ | df | P | min | Max |
| --- | --- | --- | --- | --- | --- | --- | --- | --- | --- |
| intercept | 1.023 | 0.261 | 0.523 | 1.559 |  |  | ^(1)^ | 0.713 | 1.310 |
| test day^(2)^ | -0.783 | 0.129 | -1.046 | -0.510 |  |  | ^(1)^ | -0.956 | -0.707 |
| group compos.^(4)^ | 0.340 | 0.347 | -0.360 | 1.010 |  |  | ^(1)^ | 0.050 | 0.618 |
| sex^(3)^ | -0.271 | 0.137 | -0.540 | 0.007 |  |  | ^(1)^ | -0.365 | -0.187 |
| fostered^(5)^ | -0.130 | 0.175 | -0.487 | 0.215 | 0.514 | 1 | 0.473 | -0.218 | -0.013 |
| birth wgt.^(6)^ | 0.040 | 0.069 | -0.096 | 0.179 | 0.331 | 1 | 0.565 | -0.002 | 0.104 |
| test day:sex | -0.166 | 0.088 | -0.330 | 0.010 | 3.511 | 1 | 0.061 | -0.221 | -0.103 |
| test day:group compos. | 0.345 | 0.168 | 0.007 | 0.682 | 3.420 | 1 | 0.064 | 0.220 | 0.513 |

^(1)^ not indicated because of having a very limited interpretation

^(2)^ z-transformed to a mean of zero and a standard deviation (sd) of one; mean and sd of the original variable were, 5.000 and 2.584, respectively

^(3)^ dummy coded with female being the reference category

^(4)^ dummy coded with 'mixed' being the reference category

^(5)^ dummy coded with 'no' being the reference category

^(6)^ z-transformed to a mean of zero and a standard deviation (sd) of one; mean and sd of the original variable were, 6.873 and 1.538, respectively.
